# Examinations of tRNA Range of Motion Using Simulations of Cryo-EM Microscopy and X-Ray Data

**DOI:** 10.1155/2011/219515

**Published:** 2011-03-28

**Authors:** Thomas R. Caulfield, Batsal Devkota, Geoffrey C. Rollins

**Affiliations:** ^1^School of Chemistry & Biochemistry, Georgia Institute of Technology, 901 Atlantic Avenue, Atlanta, GA 30332-0230, USA; ^2^School of Biology, Georgia Institute of Technology, 901 Atlantic Avenue, Atlanta, GA 30332-0230, USA

## Abstract

We examined tRNA flexibility using a combination of steered and unbiased molecular dynamics simulations. Using Maxwell's demon algorithm, molecular dynamics was used to steer X-ray structure data toward that from an alternative state obtained from cryogenic-electron microscopy density maps. Thus, we were able to fit X-ray structures of tRNA onto cryogenic-electron microscopy density maps for hybrid states of tRNA. Additionally, we employed both Maxwell's demon molecular dynamics simulations and unbiased simulation methods to identify possible ribosome-tRNA contact areas where the ribosome may discriminate tRNAs during translation. Herein, we collected >500 ns of simulation data to assess the global range of motion for tRNAs. Biased simulations can be used to steer between known conformational stop points, while unbiased simulations allow for a general testing of conformational space previously unexplored. The unbiased molecular dynamics data describes the global conformational changes of tRNA on a sub-microsecond time scale for comparison with steered data. Additionally, the unbiased molecular dynamics data was used to identify putative contacts between tRNA and the ribosome during the accommodation step of translation. We found that the primary contact regions were H71 and H92 of the 50S subunit and ribosomal proteins L14 and L16.

## 1. Introduction

tRNA is a key component for protein synthesis in the cell. tRNA delivers amino acids to the ribosome, where they are incorporated to the growing nascent polypeptide chains. The characteristic L-shaped tertiary structure of tRNA is intimately related to its function, and it has intrigued investigators for decades [1, 2].

**Figure 1:**
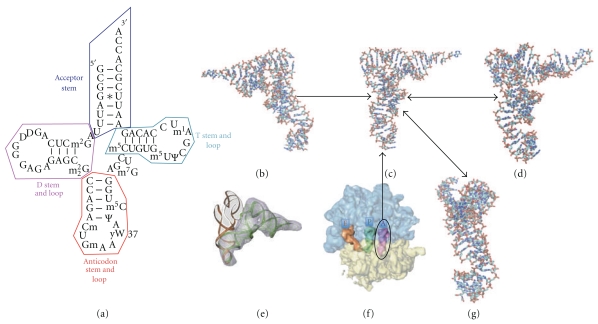
tRNA Conformations. Both kinked structures for tRNA^Phe^ (PDB code: 1EHZ) and the hybrid state for cryo-EM densities of tRNA are shown. The different orientations of tRNA depict the unique differences caused from kinking or twisting (A/T and P/E states, resp.). The arrows depict the transition from A/T state to A-site (A/A state) to P-site and P/E hybrid state. (a) Secondary structure for Phe-tRNA^Phe^ (1EHZ) [33]. (b) Structure of kinked A/T-tRNA, as it enters the ribosome (pre-accommodation) [13]. (c) A-site tRNA after accommodation occurs (post accommodation). (d) Hybrid P/E-tRNA following peptidyl transfer. (e) Comparison of A-site tRNA and kinked A/T-tRNA from modeling with cryo-EM density. (f) Position of tRNA in A-site indicated in cryo-EM density. (g) Conformation sampled from MD demonstrating an experimentally unidentified twisted conformation for tRNA^Phe^.

With the first high-resolution crystal structure for tRNA [1, 2], it was suggested that the molecule may possess a flexible hinge between the D-stem and the anticodon stem (Figure 1). Figure S6 (see Figure in Supplementary Material available online at doi: 10.1155/2011/219515) shows a diagrammed version of tRNA for illustration. The interarm consists of the hinge formed between the acceptor stem and the anticodon stem (Supplementary Figure S6). Figure 1(a) schematic shows a two-dimensional layout for the RNA nucleotides that account for the acceptor stem, anticodon stem, D-stem, and T-stem. Early light scattering experiments were interpreted in terms of bending motions between the two arms of the L-shaped tRNA that were thought to facilitate functional flexibility [3]. tRNA binds to aminoacyl tRNA synthetases, elongation factors, as well as different sites on the ribosome. 

The first concrete experimental evidence of tRNA flexibility came with determination of the tRNA^Asp^ crystal structure for which an interarm bend angle of approximately 89° was observed, larger than that observed in tRNA^Phe^ [1, 4–9]. Recently, a larger conformational difference was demonstrated using cryo-electron microscopy (cryo-EM) density maps for the kirromycin-stalled state of the ribosome, which has two tRNAs bound at the A and P sites of the small subunit [10–15]. In the cryo-EM-derived model, the incoming tRNA is shown bound in a preaccommodation state called “A/T” (Figures 1(b) and 1(e)). The cryo-EM-derived model shows a notable kink between the D-stem and the anticodon stem, where it was postulated that this might indicate that a loaded spring-like action is possible that would drive tRNA through accommodation to the A/A state [10, 13, 15].

tRNA flexibility has been a motivating topic for early molecular modeling studies on tRNA [16–18]. Prior modeling studies of tRNA have examined B-factor and H-bonding correlation with X-ray data [17–19]. More recently, large-scale tRNA motions through the ribosome were studied using biased molecular dynamics simulations [20, 21]. Another recent study focused on the structure and dynamics of a fragment of tRNA^Ala^ [22].

MD simulations have been successfully characterized as structural dynamics, base pairing, specific hydration, cation binding, and electrostatic potential distributions, including mutagenesis [20, 23–30]. Sponer et al. studied the structural dynamics, elasticity, and deformability of helix 44 (H44) from the small subunit 16S rRNA, finding that H44 has an intrinsic ability to deform on the nanosecond timescale [31]. They also observed “bulge-induced switches” in nucleic bases 1466C, 1431C, and 1467C that formed at a cost of approximately 5–7 kcal/mol [28, 32].

In the present study, we report global range of motion for tRNA derived from molecular dynamics simulations. The simulation method was a novel application first reporting the conjunction of X-ray data driven toward that of the shape given from the cryo-EM density map (Biophysical Society Meeting 2006 and 2007) [34]. We assembled a database of specific similar tRNA structures from the PDB with common stems. Using the database, insights into the modes global motion of tRNA were examined, which we then compared with our simulations. We collected two kinds of simulation data. The first type was derived used a biasing method called Maxwell's demon molecular dynamics (MdMD), which is used to flexibly fit a high-resolution crystal structure of tRNA toward that of a cryo-EM density maps for other conformations of tRNA. The second sort of simulations was an unbiased, long-running simulation of aa-tRNA. Some of the unbiased simulations included a tethered or restrained anticodon, which mimics the hydrogen bonding of the anticodon to mRNA during translation. Post simulation, we docked these tethered tRNA trajectories into a static model of the ribosome to identify putative contact regions between tRNA and the ribosome.

## 2. Methods

### 2.1. MD Protocols

Multiple simulations of aa-tRNAs were completed using NAMD version 2.62 with the CHARMM27 force field, and then the simulation was conducted in duplicate using NAMD version 2.62 with the AMBER force field [35–39]. Using duplicate simulations under different force fields allowed us to account for differences between force fields and test for differences on flexibility. A typical tRNA simulation was comprised of a water box containing between 60,000 and 100,000 TIP3P water molecules that were added around the tRNA to provide a depth of 12 to 18 Å from the edge of the molecule [40]. Starting structures for the tRNA consisted of tRNAs in the literature [9, 13, 16, 23, 33], and cryo-EM densities were given directly from our collaborator (J. Frank, personal communication). The cryo-EM densities used for simulation are obtained from isolated *E. coli* ribosome microscopy data of tRNA/RNA/ribosome complexes.

Several tRNA simulations were run only using neutralizing Na^+^ ions. These were initially placed using the Xleap module of AMBER9 at the positions of the lowest electrostatic potential. In one case, we neutralized with 76 Na^+^ counterions to the tRNA. In another case, we neutralized with counterions and then created a solvent with 150 mM Na^+^ Cl^−^ to recreate physiological strength. We observed similar tRNA flexibility in both cases.

The AMBER force field parameters for the naturally occurring modified nucleosides of RNA were obtained from the web-server at the lab of SantaLucia and from the Sanbonmatsu lab (personal communication) [41]. We used the following van der Waals parameters for Na^+^: radius 1.868 Å and well depth of 0.00277 kcal/mol [42]. 

Simulations were carried out using the particle mesh Ewald technique (PME) with repeating boundary conditions in a box approximately 70–105 Å^3^ with 9 Å nonbonded cutoff [43], using SHAKE with a 2 fs timestep [44]. Pre-equilibration was started with 10,000 steps of minimization followed by 250 ps of heating under MD, with the atomic positions of tRNA fixed. Then, two cycles of minimization (1000 steps each) and heating (200 ps) were carried out with restraints of 10 and 5 kcal/(mol*·*Å^2^) applied to all tRNA atoms. Then, 1000-steps of minimization were performed with solute restraints reduced by 1 kcal/(mol*·*Å^2^). Then, 200 ps of unrestrained MD were carried out, and the system was slowly heated from 1 to 310 K. The production MD runs were carried out with constant pressure boundary conditions (relaxation time of 1.0 ps). A constant temperature of 300 K was maintained using the Berendsen weak-coupling algorithm with a time constant of 1.0 ps. SHAKE constraints were applied to all hydrogen atoms to eliminate X-H vibrations, which yielded a longer simulation time step (2 fs). Our method of equilibration and production simulation is similar to protocol in the literature [30, 31].

Translational and rotational center-of-mass motions (COMs) were initially removed. Periodically, simulations were interrupted to have the COM removed again by a subtraction of velocities to account for the “flying ice-cube” effect [45]. Following the simulation, the individual frames were superposed back to the origin, to remove rotation and translation effects.

### 2.2. MdMD Protocols

In the MdMD algorithm, we run a short “sprint” of MD (typically ranging from 50 fs to 5 ps) [34]. We then compute the value of a global progress variable. If the MD sprint has moved the system toward the goal value of the progress variable, we save the state of the system and carry out another MD sprint. If the system has moved in the wrong direction with respective to its goal, we reset the system to the last archived state, reset the velocities, and repeat the MD sprint. In this way, we only retain the MD steps that move the system toward its goal. We repeat this cycle of MD sprints until the system reaches its goal. In this study, we defined the global progress variable to be the cross-correlation between the electron density of the simulation structure and the experimental density from the cryo-EM density map. We rejected MD sprints that decreased the cross-correlation.

### 2.3. Cryo-EM Fitting Using MdMD

This application of MdMD allows the user to alter the shape of X-ray crystallographic structures to match the cryogenic-electron microscopic data, which may present an alternative conformation of the structure. In doing so, the cryo-EM density can drive the MD toward an unknown conformation. By automating this process, human biases and errors are minimized for the model making process. The first step is conversion of the MD all-atom structure into a representative low-resolution cryo-EM density, and the second step is the comparison of the MD-generated density to the target cryo-EM density. If the correlation between the MD density and the target density increases, then the iteration is saved and the process continues; otherwise the structure returns to the prior state and another MD sprint is carried out. We verified the correlation of the densities using both SPIDER and NMFF [46–48]. Our MdMD method of flexible cryo-EM fitting, as described herein, is cited within the Flexible Fitting program (FFMD) [34, 49]. In the FFMD program [49], a gradient is applied to the potential based upon the cross-correlation coefficient (CC), whereas in the MdMD-type cryoEM fitting algorithm there are external entropic forces applied in selection.

The CC represents the numerical correlation between the electron density of the target cryo-EM density map and the simulated density map from the current MD structure. The CC is defined as follows:

(1) $$\text{ C.C.}=\frac{\sum_{ijk}^{}\rho^{\exp\;}\left(i,j,k\right)\rho^{\text{sim}}\left(i,j,k\right)}{\sqrt{\sum_{ijk}^{}\rho^{\exp\;}{\left(i,j,k\right)}^{2}\sum_{ijk}^{}\rho^{\text{sim}}{\left(i,j,k\right)}^{2}}},$$

where *ρ* ^Exp^ and *ρ* ^Sim^ are the experimental and simulated densities for the voxels (*i*, *j*, *k*). The simulated density map is generated from the MD structure, using a Gaussian function on every atom, and then integrating for each of these atom in all of the given voxels, as determined by its set of atomic coordinates (*x*_*n*_, *y*_*n*_, *z*_*n*_), where *ρ* ^Sim^(*i*, *j*, *k*) = Σ∫*V*_*ijk*_ *dxdydz* for *N* atoms, and (*i*, *j*, *k*) being a given voxel of *g*(*x*, *y*, *z*, *x*_*n*_, *y*_*n*_, *z*_*n*_). Using the Gaussian, *g*(*x*, *y*, *z*, *x*_*n*_, *y*_*n*_, *z*_*n*_) = exp [−3/2*σ* ^2^((*x*−*x*_*n*_)^2^ + (*y*−*y*_*n*_)^2^ + (*z* − *z*_*n*_)^2^)], with sigma as a parameter of the resolution used. We use a 2*σ* cutoff for the resolution in our simulated maps in order to approximate the experimental resolution. This method of calculating a density is similar to that used to generate electron densities by methods in the literature; however we also tested the program SPIDER to generate simulated electron densities [46, 47]. SPIDER only differed slightly in the amount of CPU time to calculate a correlation and a slight drop in overall efficiency.

## 3. Results

### 3.1. Database of Crystal Structures

**Figure 2:**
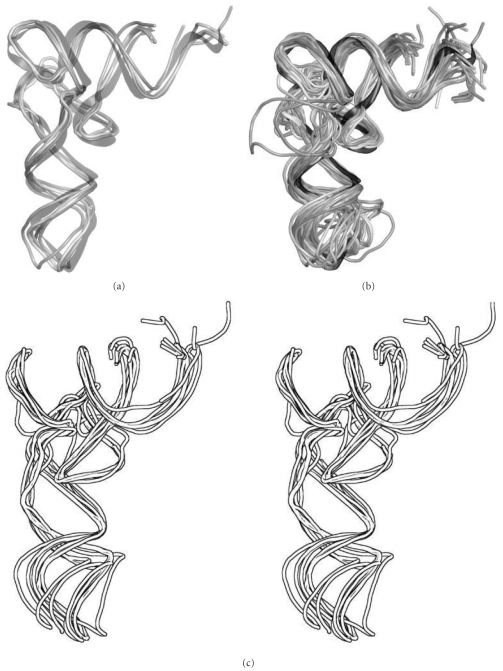
tRNA Catalog. (a) Six crystal structures of unbound Phe-tRNAPhe. The ribbon in the middle is from 1EHZ. (b) Crystal structures (41) of tRNA free and in complexes, including synthetases and ribosomal (Table S1) aligned at the acceptor stems and the anticodon stems. The ribbon in the middle is from 1EHZ. (c) Phe-tRNAPhe crystal structures (6) aligned at the acceptor stem (shown in divergent stereo), thus illustrating the range of motion at the anticodon stem loops, and notice the closely aligned acceptor stem regions.

We compiled a set of tRNA crystal structures from structural databases to quantify any tRNA flexibility from experimental data. The Nucleic Acid Database and the Protein Data Bank RCSB [50, 51] contain over 80 structures of tRNAs alone or in complexes with other molecules at a resolution of 3.3 Å or better. For the study presented we focused on a subset that were in good alignment with tRNA^Phe^. Thus, thirty-four of the crystal structures are tRNA-protein complexes. The remaining six are free, unbound tRNA structures. Most of the complexes are aminoacyl-tRNA synthetases (32 structures), but there is also one cocrystal of tRNA^Glu^ with its amidotransferase, and another between tRNA^fMet^ and its formyltransferase (Figure 2(b)). Of the isolated tRNA structures, six are unique and of the highest available resolution, while four are distinct: Asp from *E. coli* and *S. cerevisae*, Lys from *B. taurus*, Phe from *S. cerevisae* and *T. thermophilus*, and fMet from *E. coli* and *S. cerevisae* (Figure 2(b)) [3]. tRNAs for fMet and 16 other amino acids are represented (Figures 2(a) and 2(b)). Supplementary Table S1 summarizes the crystal structures that we examined. Figure 2 demonstrates the conformational variability among the full set of structures, and Figure 2(c) shows the variability in just phenylalanine-tRNA^Phe^.

We used the interarm angle between the anticodon stem and the acceptor stem as a measure of flexibility. There were eight species of tRNA for which multiple X-ray crystal structures were available: Asp, Glu, Gln, Met, Phe, Trp, Tyr, and Val. All eight of these had similar ranges of interarm angles, somewhere between 70° and 100°, which suggests that interarm flexibility is an intrinsic feature of the topology of tRNA and does not depend strongly on the amino acid bound to the acceptor stem (Supplementary Table S1).

**Figure 3:**
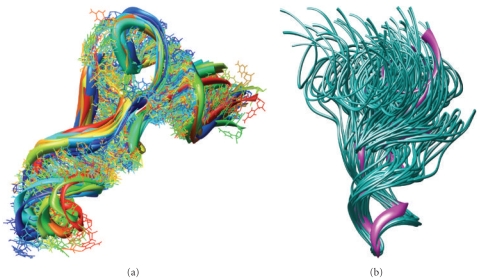
Jittergram of kinked A/T-tRNA from molecular dynamics simulations. (a) Using multiple unbiased molecular dynamics for kinked A/T-tRNA, we generated a set of structures. These are superposed based on all P-atoms found in the backbone of the stem regions. This orientation optimally shows the alignment of the stems and the variation at the acceptor stem loop and anticodon stem loop. The different tRNA colors help distinguish the different conformations. (b) Illustrated in cyan as a series of snapshots is a simulation for kinked A/T-tRNA using free molecular dynamics with tethering constraints at the anticodon atoms (H-bond atoms of nucleotides 34, 35, 36), which was run for >10 ns. We used tethering (harmonic constraints) at the anticodon atoms to mimic base pairing at the codon. This data was used for the contact map (Figure 5). This orientation best shows the wide range of motion that the tRNA covers (over 70 Å from beginning to end for the 3′CCA end).

In addition, we computed the RMSDs of the crystal structures, relative to 1EHZ tRNA^Phe^, and found a range from 0.5 Å to approximately 6 Å (Supplementary Table S1) for superposition of the main stems. Consequently, on a residue-by-residue basis, the greatest regions of flexibility were the 3′-CCA end of tRNA and the anticodon stem-loop (ASL) (Figure 3(a), Supplementary Movies S1, S2, S5).

### 3.2. Fitting to Cryo-EM Maps via MdMD

Cryo-EM reconstructions of tRNA bound to the 70S *E. coli* ribosome at different steps in the translation-elongation cycle were examined [14, 52]. From the reconstructions, it is possible to identify subtle conformational changes in tRNA (Figure 1(e)). For example, at a resolution of 7.8 Å a cryo-EM density map can reveal the helical structure of tRNA for the hybrid “A/T” state, which is deformed at the anticodon stem arm through interactions with the D-stem loop residues 26, 44, and 45 of phenylalanine-tRNA^Phe^ (Figure 1(e)).

We examined four distinct tRNA conformations derived from cryo-EM density maps. These tRNA conformations included the A, P, A/T, and P/E states. Using Maxwell's demon Molecular Dynamics (MdMD), the starting structure of the native tRNA structures (1EHZ) is driven into conformations that match the cryo-EM data based upon a cross-correlation calculation between a theoretical density for the modeled structure and the experimental cryo-EM density. MdMD is derived from existing methods in the literature for biasing simulations [53]. However, in addition to having an entropic penalty for directional control, there is an adaptive component that dictates the amount of sampling time per interval of MD [34]. A brief explanation of MdMD consists of the following iterative steps: (1) a short interval of MD simulation (MD sprint) is completed, (2) the MD is paused and a “check” of some user defined variable is assessed, which determines a numerical value for the *progress* of the variable, and (3) the algorithm either accepts or rejects the result of the progress variable, which is based on the current value of the progress variable plus several previous archived states. The adaptive component will accept an MD sprint and increase or decrease the amount of sampling time for the next MD sprint based on the new value for the progress variable. Likewise, the sampling size may shrink if the success criteria opposite the goal. 

The steps are archived into a single trajectory based upon the outcome of the progress variable. During the MD sprint the variable is free to fluctuate into space that would otherwise be forbidden by the Maxwell demon. 

In the case of cryoEM-fitting with MdMD, our progress variable is the cross-correlation between the electron density of the simulation structure and the experimental density from cryo-EM, where we rejected MD sprints that decrease the cross-correlation.

**Figure 4:**
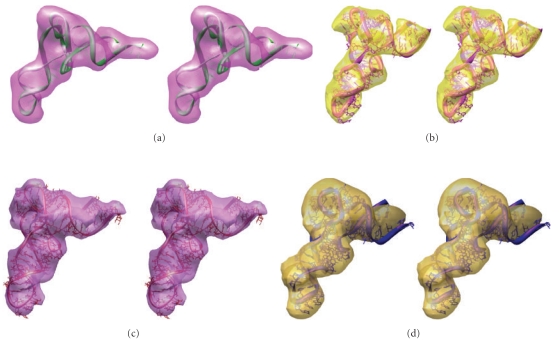
Cryogenic-electron microscopy fitting using Maxwell's demon Molecular Dynamics for tRNAs A/T, A, P, and P/E is shown. All tRNA atomic models are shown in ribbons/CPK, while densities are as solid. (a) A/T-site structure for tRNA fit to density, cross-correlation coefficient (CCC) of 95% between the structures theoretical density and the cryo-EM experimental density (Divergent stereo). (b) A-site structure for tRNA fit to density with a CCC of 83% (Divergent stereo). (c) P-site structure for tRNA with a CCC of 86% (Divergent stereo). (d) P/E-site structure with a CCC >90% (Divergent stereo).

Using MdMD, we obtained pathway transitions from the crystal structure state (1EHZ) to the cryo-EM states A, P, A/T, and P/E using less than 10 nanoseconds of molecular dynamic simulation (Supplementary Movies S4, S6). From these simulations, the ensembles of structures are consistent with the density distributions from the cryo-EM density maps. Figure 4 shows the quality of fit for the four structures. Supplementary Table S2 identifies the cryo-EM density maps of each structure and gives the RMSD of each structure relative to the crystal structure of yeast tRNA^Phe^ (PDB code: 1EHZ) [33]. All-atom root mean-squared deviation (RMSD) analyses between the crystal structure and regions from our molecular dynamics model are <2 Å at these superposed regions, indicating that the fine structure was maintained. Using MdMD, the A-site conformation can be forced rapidly to the kinked conformation required for the A/T state. The kinked structure generated using MdMD is within 1.0 Å RMSD of the original manually modeled structure of the A/T tRNA (Supplementary Movie S6) [13].

The cross correlations between the fit structures and the maps give a good indication that the fit structures are not deformed. Specifically, the pathways between the fit structures reveal the transitional motion, dynamically, between modes of tRNA: from A/T to A, from A to P, and from P to P/E, respectively, exploring stochastically reversible excursions along a tRNA pathway between experimentally verified states [54].

### 3.3. Testing tRNA Motion Using Unbiased Molecular Dynamics

We carried out two sets of unbiased MD simulations on tRNA: (1) We ran ~0.5 *μ*s of simulations starting from the crystal structure of yeast tRNA^Phe^ (1EHZ) to examine the inherent flexibility of tRNA. (2) We asked whether tRNA in the twisted A/T conformation would spontaneously move toward the native crystal structure for yeast tRNA^Phe^ using unbiased MD [13]. In this case, the anticodon stem must untwist to find the native state. We found that A/T tRNA does so quite rapidly (Supplementary Movie S1). The unkinking occurs below the hinge, toward the anticodon. 

We repeated this simulation with a tethered anticodon to mimic the hydrogen bonding with mRNA. We imposed restraints on anticodon nucleotides 34, 35, and 36 (1 to 10 kcal/(mol∗Å^2^) per hydrogen bonding atom). One would expect that this tether would result in dampening of tRNA motion, because the D-loop, T-loop, and acceptor-stem must rotate in order to relieve the twist in the anticodon stem. This simulates the effect of being bound to the ribosome (Supplementary Movie S2). This situation is similar to the simulation of Sanbonmatsu et al., except that there is no ribosome present to hinder swinging, and no biasing forces were used [20, 54].

From our unbiased MD simulations, we found that tRNA^Phe^ unkinked in the 2 ns following equilibration, when the anticodon atoms were unrestrained. However, when the anticodon was restrained, unkinking occurred between 6 and 10 ns. We also found that simulations of the A/T to the A/A state provide an approximate range for tRNA swinging-type motion that agrees with the structural data in our database of crystal structures.

Our results suggest that tRNA deforms quite readily at the anticodon stem from an initial free tRNA state. We found that (1) the kinked A/T structure's trajectory converged toward the crystal structure of tRNA^Phe^ (Figure 6, Supplementary Figure S2), and (2) tRNA^Phe^ (1EHZ) relaxed under unbiased MD to an equilibrated state [13, 33]. This relaxed structure for tRNA^Phe^ sampled regions of conformational space that were similar to those sampled by the kinked A/T structure. The trajectory of the kinked A/T and the trajectory of the native tRNA^Phe^ structure overlapped in conformational space around 1.4 ns, 2.7 ns, and after 5 ns (Supplementary Figure S2). The RMSD matrix plot identifies several well-populated regions of conformational space between the A/T and A/A states (Figure S2). Also, we were able to rapidly drive the crystal structure of tRNA^Phe^ (1EHZ) to form the kinked A/T structure with MdMD (~8 ns) (Supplementary Movie S3, Figure S2). Both force fields were successful in achieving unkinking, but the helical parameters of base pairing were better modeled with the amber force field [36, 37, 39].

**Figure 5:**
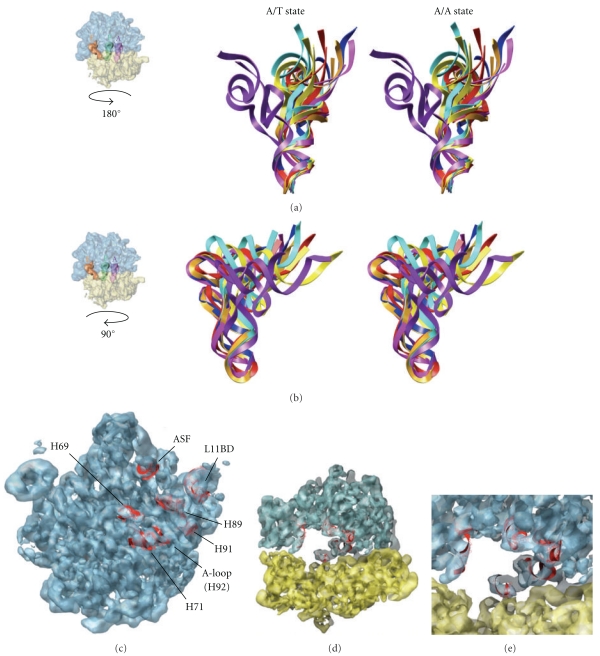
Various snapshots from multiple simulations of A/T-tRNA and native tRNA are shown, where the time sampled over is >200 ns (Supplementary Movie S1). (a) A/T-site tRNA (shown in purple) moves toward the A-site tRNA (red) which is shown in reverse view of the ribosome density, to illustrate the >70 Å motion covered by the 3′-CCA acceptor end. The tRNA shows an oscillation about the A-site tRNA structure (red) (Divergent stereo). (b) *Side view* of Figure 5(a) (Divergent stereo). (c) Contact map for tRNA trajectory superposed with the ribosome. (d) The side view panel on right shows the accommodation corridor for the A/T-tRNA in the ribosome. The A/T-tRNA has to make a snug fit because of the narrow space. (e) The lower right panel is zoomed in closer to illustrate the interaction areas.

Figures 3(a) and 3(b) and Figures 5(a) and 5(b) show the range-of-motion accessible to tRNA during multiple 100+ ns simulations. In Figure 3(a), we aligned the trajectories of structures at their center-of-mass. The anticodon stem loop region fluctuated 7–10 Å, while the 3′-CCA end of the acceptor stem fluctuated 13–15 Å (Movies S1, S2). Figure 3(b) shows a progressive pathway for tRNA with a constrained anticodon during simulations of unkinking from the A/T state to the A/A state. The aminoacyl-3′-CCA tip of the acceptor stem moved approximately 70 Å from start to finish. Figures 5(a) and 5(b) show several snapshots from unbiased simulations. They appear to fluctuate around a structure that resembles the A-site structure of tRNA. The A-site structure also resembles the crystal structure of tRNA^Phe^ (1EHZ). The native tRNA and A-site tRNA exhibited an average RMSD of 4 Å among all the tRNAs and 2.2 Å for the Phenylalanine-tRNA^Phe^ structure. The A/T structure is shown for reference in purple.

**Figure 6:**
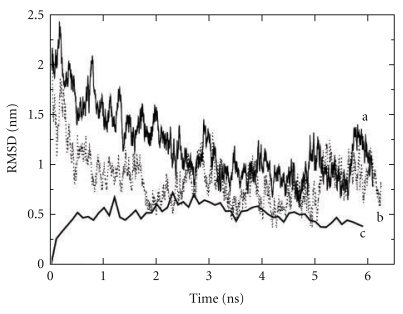
RMSD graph for three types of tRNA simulations presented in this study. This graph represents the root mean square deviation between the simulation of tRNA^Phe^ with that of the crystal structure for tRNA^Phe^ (1EHZ) [33]. As time progresses the initial tRNA^Phe^ relaxes under the force field and the biased and unbiased states of tRNA progress toward the crystal form. (a) “Line a” represents the constrained anticodon MD simulation of the A/T state kinked model, which converges to the A-site ensemble after 4 ns. (b) “Line b” represents the free MD simulation of the A/T state kinked model that converges much quicker to the A-site ensemble of structures (2 ns). (c) “Line c” represents the free molecular dynamics simulation for the A-site crystal structure model, which moves from the starting structure to an average ensemble around 4 Å RMSD from the original as it relaxes under MD.

The RMSD values from multiple simulations of kinked and unkinked tRNA, as well as native tRNA, show that these different conformations often converge within 10 ns, indicating that the favored conformation in all cases occurs in a region of conformational space similar to the relaxed native structure (Figure 6). For all simulations addressed here, RMSD convergence occurs during the initial 10 ns and then again periodically. Here, convergence is defined as an RMSD below 3 Å. Longer sampling (>100 ns) showed that twisted conformations similar to the hybrid states like P/E or A/T are accessible from the native form of tRNA (1EHZ). Our long trajectories also sampled many of the conformations found in our set of tRNA crystal structures.

### 3.4. A Map of Ribosomal Contact Points from Simulation Data Overlap

We docked the unbiased MD simulation of tethered tRNA into the ribosome in order to study contacts between tRNA and the 70 S *E. coli* ribosome. We compiled the results of our simulations (0.5 *μ*s in total) into a contact map (Figures 5(c) and 5(d)). Our definition of a contact is based on C-alpha-to-RNA base distances and base-to-base distances within a 4 Å cutoff. The map shows that tRNA interacts with ribosomal proteins L14 and L16 as well as H71 and H92 of the 50S subunit during accommodation. These areas may act to filter out less energetically kinked tRNAs during accommodation. If near-cognate tRNAs are less kinked than cognate tRNAs, this process might filter them out. There is significant agreement between our proposed contact map and the transition pathways observed in the literature [20, 21, 54].

Recent data presented in the literature demonstrates a low-resolution structure for the 80S P-site tRNA that suggests a state in which the anticodon stem-loop is significantly bent [55]. This occurs during programmed ribosomal frame shifting, when the ribosome is subverted into a frame shift by maintaining the 3′-CCA acceptor end bound, while the anticodon takes a −1 step, thus placing the message into a new reading frame [55]. We observed similar deformations during our simulations (Movie S6). Additionally, during the formation of the kinked A/T state, we observed deformations of the D-loop relative to the acceptor stem (Movies S1, S5).

## 4. Conclusions

Our simulations of tRNA support a complex flexible hinge motion between the anticodon stem and acceptor stem with a characteristic relaxation time on the nanosecond timescale. The hinge is centered on nucleotides 26, 44, and 45, and additionally there are complex stem deformations visible in the dynamics movies. In simulations where the anticodon was tethered, which mimic hydrogen bonding to the mRNA codon during translation, we found that the correlation time for rotational diffusion of tRNA about the fixed codon-anticodon duplex was approximately 10 ns. We found that tethering the anticodon bases in space resulted in diffusion being driven by an unkinking force, which was able to swing the entire tRNA 3′-terminus along a trajectory mimicking tRNA accommodation in the ribosome during translation. As the A/T-tRNA moved away from the kinked state, it progressed into a range of conformations similar to those found in other catalogued crystal structures.

Our results confirm that tRNA has a stochastic molecular spring-like motion from the biasing method [52, 54], and from the unbiased simulations a nonlinear Brownian type motion from the A/T-structure toward the A-site state model, which matches the free tRNA^Phe^ crystal structure (1EHZ). Moreover, all tRNAs tested possess an intrinsic ability to use flexibility to achieve a kinked state. Hybrid states of tRNA might be utilized by the ribosome to load the spring as a result of translocation. Thus, the ribosome may be inducing tRNA into the next position, twisting it into the hybrid state. Following this loading process, tRNA would untwist via a stochastic spring-like motion. 

Here, we report that a Maxwell demon-type algorithm that acts externally to the potential one may utilize experimental cryo-EM densities to “drive” molecular dynamics simulations into preferred conformations. This result is promising for future work to obtain transition pathways. We found that our biased MdMD method can sample multiple hybrid conformations of tRNA that have been experimentally observed from cryo-EM and X-ray crystallography, thus bridging between two experimental methods. 

Finally, we constructed a putative ribosomal contact map from the microsecond dynamics of tRNA bound to the ribosome. We observed contacts with H71 and H92 of the 50S subunit and ribosomal proteins L14 and L16. It is possible that these areas of the ribosome act to filter near-cognate tRNA in a test of anticodon base pairing during accommodation.

## Supplementary Material

The supplementary material contains additional material for methods and measurements conducted. The contents include: examples of the range of motion possible using the biasing entropic method (MdMD), matrix plots for anticodons over simulation time from different starting points that examine relatedness of the tRNAs, the overall extension-compactness of the tRNA from a Rg and RMSD calculations with and without the presence of counterions like Mg++, and graph of MdMD convergence compared to unbiased MD. Also included are a schematic of the tRNA angles and a flowchart of the MdMD algorithm.
